# Knowledge about tuberculosis, treatment adherence and outcome among ambulatory patients with drug-sensitive tuberculosis in two directly-observed treatment centres in Southwest Nigeria

**DOI:** 10.1186/s12889-021-10698-9

**Published:** 2021-04-07

**Authors:** Rasaq Adisa, Teju T. Ayandokun, Olusoji M. Ige

**Affiliations:** 1 Department of Clinical Pharmacy and Pharmacy Administration, Faculty of Pharmacy, University of Ibadan, Ibadan, Nigeria; 2 Pulmonary/Chest unit, Department of Medicine, College of Medicine, University of Ibadan and University College Hospital, Ibadan, Nigeria

**Keywords:** Drug-sensitive tuberculosis, Directly observed treatment short-course, Knowledge, Treatment outcome, Nigeria

## Abstract

**Background:**

Tuberculosis (TB) remains one of the most common infectious diseases worldwide. Although TB is curable provided the treatment commenced quickly, appropriately and uninterrupted throughout TB treatment duration. However, high default rate, treatment interruption and therapy non-adherence coupled with inadequate disease knowledge significantly contribute to poor TB treatment outcome, especially in developing countries. This study therefore assessed knowledge about TB and possible reasons for treatment non-adherence among drug-sensitive TB (DS-TB) patients, as well as evaluated treatment outcomes for the DS-TB managed within a 5-year period.

**Methods:**

A mixed-method design comprising a cross-sectional questionnaire-guided survey among 140-ambulatory DS-TB patients from January–March 2019, and a retrospective review of medical-records of DS-TB managed from 2013 to 2017 in two WHO-certified TB directly-observed-treatment centres. Data were summarized using descriptive statistics, while categorical variables were evaluated with Chi-square at *p* < 0.05.

**Results:**

Among the prospective DS-TB patients, males were 77(55.0%) and females were 63(45.0%). Most (63;45.0%) belonged to ages 18-34 years. A substantial proportion knew that TB is curable (137;97.9%) and transmittable (128;91.4%), while 107(46.1%) accurately cited coughing without covering the mouth as a principal mode of transmission. Only 10(4.0%) mentioned adherence to TB medications as a measure to prevent transmission. Inaccessibility to healthcare facility (33;55.0%) and pill-burden (10,16.7%) were topmost reasons for TB treatment non-adherence. Of the 2262-DS-TB patients whose treatment outcomes were evaluated, 1211(53.5%) were cured, 580(25.6%) had treatment completed, 240(10.6%) defaulted, 54(2.3%) failed treatment and 177(7.8%) died. Overall, the treatment success rate within the 5-year period ranged from 77.4 to 81.9%.

**Conclusions:**

Knowledge about TB among the prospective DS-TB patients is relatively high, especially with respect to modes of TB transmission and preventive measures, but a sizeable number lacks the understanding of ensuring optimal TB medication-adherence to prevent TB transmission. Inaccessibility to healthcare facility largely accounts for treatment non-adherence. Outcomes of treatment within the 5-year period show that nearly half were cured, while almost one-tenth died. Overall treatment success rate is about 12% below the WHO-defined target. There is generally a need for concerned stakeholders to step-up efforts in ensuring consistent TB enlightenment, while improving access to TB care is essential for better treatment outcome.

**Supplementary Information:**

The online version contains supplementary material available at 10.1186/s12889-021-10698-9.

## Background

Tuberculosis (TB) remains one of the most common infectious diseases worldwide [1–3]. It is estimated that about 10 million people were infected with TB in 2017 with 1.3 million deaths among HIV negative people and an additional 350,000 deaths among HIV positive [4, 5]. Tuberculosis incidence rates in Africa have been decreasing at a rate of 4% per year between 2013 and 2017, however, TB incidence rates in Nigeria have remained steady from 2000 through 2017 [4, 6]. About 429,000 people in Nigeria have TB each year, while the total TB incidence rate was reported as 219 per 100,000 population [1, 4, 6]. Nigeria ranked number one in Africa and sixth globally among the 30 high TB burden countries and is also among the 14 countries in the world with the triple high burden of TB, TB/HIV and MDR-TB [1, 3, 6]. The global TB treatment success rate was reported as 85% among all new tuberculosis cases, and in Nigeria, TB treatment success rate progressed from 79 to 86% between year 2000 and 2017 [5, 7].

Although TB is curable if treatment commenced quickly, appropriately and uninterrupted throughout the 6–9 months course of treatment [1, 4, 5]. However, high default rate, treatment interruption and therapy non-adherence coupled with inadequate disease knowledge significantly contribute to poor TB treatment outcomes among TB patients [5, 7, 8]. Therefore, accurate diagnosis, use of effective anti-TB medications and optimal adherence are priority tools for minimizing morbidity and mortality, as well as mitigating the spread of TB among the population [8–11]. In Nigeria, the standard short-course therapy for all categories of drug-sensitive tuberculosis (DS-TB) comprised a 6-month regimen, with 2-month intensive phase of four medications (HRZE) viz. Isoniazid (H), Rifampicin (R), Pyrazinamide (Z) and Ethambutol (E), and a 4-month continuation phase of two medications viz. Isoniazid and Rifampicin (i.e. 2HRZE/4HR) [10, 12–15]. The treatment remained free of charge through the donor support funds, particularly the Damien foundation, and the regimen is administered daily to TB patients in the clinic under the direct supervision of healthcare workers who observe and record patient taking each TB dose (i.e. health facility-based directly observed therapy (DOT) [10, 13–15]. Also, the treatment guideline or recommendation for TB patients co-infected with HIV indicated that antiretroviral therapy (ART) should be initiated in all TB-HIV co-infected patients regardless of the CD4 cell counts, within the first 8 weeks of TB treatment (intensive phase), while HIV positive patients with profound immunosuppression (CD4 cell counts < 50 cells /mm3) should receive ART within the first 2 weeks of initiating TB treatment [5, 10, 16–18].

The directly observed therapy concept is one of the five components of DOTs strategy endorsed by the World Health Organisation to create the basis for standard TB care and management [10, 19, 20]. The DOTs strategy had been widely promoted and implemented in many developed and developing countries [10, 21]. Nigeria adopted the DOT concept in 1993, as a proactive core management approach to address non-adherence problem among TB patents [12, 14, 19]. However, despite the significant progress made in the control of TB through DOTs strategy, as well as potential advantages of DOT approach in enhancing adherence, TB has remained prevalent, while treatment outcomes and success rate still falls below the WHO defined target especially in low and middle-income countries (LMICs) including Nigeria [3, 4, 14, 22].

The WHO Global Tuberculosis Report 2020 identified the latest challenges to TB management to include equitable access to quality and timely diagnosis, prevention, treatment and care [5]. However, non-adherence to TB treatment had also been consistently recognised as a principal factor linked to poor treatment outcomes and suboptimal TB control globally [21, 23–25]. Treatment adherence among TB patients is challenging given the complexity, modest tolerability and long duration of treatment regimen currently available for both drug-susceptible and drug-resistant TB [23, 26]. Adherence to TB medications is estimated to be as low as 40% in developing countries including Nigeria [25]. Low adherence may result in failure of initial treatment, emergence of multidrug resistant tuberculosis (MDR-TB), prolonged infectiousness and poor TB treatment outcomes [23, 26–28]. In addition, TB patients who are not cured due to treatment non-adherence may pose a serious risk for individuals and community [24, 26, 29]. The WHO recommends at least 85 to 90% treatment success rate for all diagnosed TB cases [1, 7]. However, to achieve the target among TB patients, there may be a need for better understanding of the particular barriers to TB treatment adherence, as well as patients’ knowledge and experience about TB and its management [30]. This may become necessary since adherence to treatment is critical for cure of TB, as well as controlling the spread of TB infection, while minimising the development of drug resistance [27]. Also, possession of adequate knowledge of the disease may aid the uptake of TB services [28].

Though, there are studies from many developed and some developing countries that had evaluated knowledge, attitude and practice about TB, as well as barriers to TB treatment adherence [30–35]. However, most of these studies still left gaps that underscore the necessity for continuous monitoring and evaluation of patient-specific reasons for TB treatment non-adherence, while making consistent efforts to evaluate the knowledge deficits of patients about TB may be essential in finding appropriate solution to the low TB treatment success rate. This study therefore assessed knowledge about TB and the possible reasons for suboptimal treatment adherence among ambulatory drug-sensitive TB (DS-TB) patients in two WHO-certified TB-DOT hospitals in Ibadan, southwest, Nigeria. Also, the treatment outcomes documented in the medical records of DS-TB patients managed in the hospitals between 2013 and 2017 were evaluated.

## Method

### Study design

The study employed a mixed-method design comprising a prospective questionnaire-guided cross-sectional survey among DS-TB patients for eight consecutive weeks, between January and March, 2019, and a retrospective review of medical records of DS-TB patients managed within the 5-year period in the two hospitals.

### Study setting

The tuberculosis DOT clinic of the University College Hospital (UCH) and Government Chest Hospital Jericho (GCHJ) Ibadan. Both hospitals are WHO-certified TB-DOT centres of excellence, supported by Damien Foundation, Belgium, and they both have fully equipped and functional DOT clinic for management of TB patients.

### Study population

Adult outpatients (> 18 years) with DS-TB and who were registered with the TB-DOT clinic of the hospitals.

### Inclusion and exclusion criteria

All consenting DS-TB outpatients, aged over 18 years, who were on TB treatment for at least one month prior to the commencement of the study were included. Patients with MDR-TB, as well as pregnant women whose condition may necessitate adjustment of standard TB dosage regimen were excluded. Also, case notes of DS-TB patients with incomplete data, especially with respect to treatment outcomes were excluded.

### Sample size determination

Representative sample size for the study was calculated using Raosoft® sample size calculator (www.raosoft.com/samplesize.html). Eligible population of adult outpatients with DS-TB attending the TB-DOT clinic in UCH and GCHJ were estimated as 55 and 125, respectively for the 8-weeks study period. Thus, with the estimated population of 180 from both hospitals, and assumptions of 95% confidence level, 5% margin of error, as well as 50% conservative estimate to represent the proportion in the target population estimated to have a particular characteristics, a sample size of 125 was obtained. However, adjusting for 10% attrition rate, gave a target sample size of approximately 138 (rounded off to 140) patients. Subsequently, the proportion of participants recruited from each hospital was determined as follows: UCH: (55 ÷ 180) × 140 = 42.7; GCHJ: (125 ÷ 180) × 140 = 97.2. Approximately 40 patients from UCH and 100 from GCHJ were used as target sample size to guide participants’ enrolment. Also, the medical records of all DS-TB patients managed between 2013 and 2017 were selected and reviewed accordingly.

### Data collection instrument

The questionnaire and data collection form were designed by the investigators following extensive review of relevant studies [30–37], as well as previous practice experience. The prospective cross-sectional survey consisted of 25-item questions, including open-ended, closed-ended and open-ended questions with relevant prompts. The questionnaire has three sections, with the primary outcomes measured in Section A included socio-demographic characteristics such as age, sex, educational qualification, occupation and marital status, as well as clinical characteristics including duration on TB treatment, family history of TB, as well as symptoms experienced at the onset of TB infection. Section B contained questions that evaluated patients’ knowledge about TB, modes of TB transmission and suggested preventive measures, as well as TB medications and other adjuncts being taking by the patients. Section C focused largely to explore the patients about possible reason(s) for TB treatment non-adherence, side effects experienced with TB medications and reporting of such side effects to healthcare provider (See additional file 1).

The retrospective review of medical records of DS-TB patients was guided by data collection form to retrieve information on demographic characteristics especially age, sex and year of treatment. Also retrieved were disease-specific clinical parameters including sputum smear acid-fast bacilli (AFB) results and any other diagnostic options, as well as patients’ HIV status and outcomes of treatment. In this study, treatment outcomes explored for the DS-TB retrospective cohort included: cured defined as pulmonary TB patients with smear or culture negative in the last month of treatment and in at least one previous occasion; completed treatment defined as pulmonary TB patients with smear or culture negative in the last month of treatment and in at least one previous occasion; failed treatment defined as a positive sputum smear or culture at the month 5 or later during treatment; defaulted defined as an interruption of TB treatment for 2 or more consecutive months; and died defined as TB patients who dies for any reason before starting or during the course of treatment. The definitions were in accordance with the WHO TB treatment guidelines and National Tuberculosis and Leprosy Control Programme (NTBLCP) classifications of TB treatment outcomes [7, 10, 12]. In our study, transferred out was defined as TB patients for whom no treatment was assigned, and which include those transferred out to another facility or treatment unit. The transferred out patients were only captured in the review and documentation, but were not considered as part of treatment outcomes.

Also, successful treatment was defined as the sum of TB patients who were cured and those who completed treatment, while unsuccessful treatment was defined as the sum total of TB patients who defaulted, failed treatment and died [7, 10, 12].

### Pre-test and validation of data collection instrument

The questionnaire was assessed for content validity by a panel of consultant pulmonologist working in each of the DOT clinics and a clinical pharmacist in the academia. Subsequently, a pre-test of the instrument was done among 14 randomly selected TB patients from the GCHJ, representing 10% of the total number of patients enrolled for the study. These patients were excluded from the main study. Feedback from pre-test and validity assessment led to minor modifications in the questionnaire including some closed-ended questions which were rephrased in open-ended format with relevant prompts to guide patients’ opinion.

### Sampling and recruitment procedure

Eligible DS-TB patients were consecutively enrolled on daily TB-DOT clinic of the hospitals while waiting for their turn of directly observed TB medication-taking or consultation with the attending healthcare provider. Individual patient was courteously approached by the investigators on the daily DOT clinic while strictly observing the TB precautionary measures. Procedure and objectives of the study were comprehensively explained to participants, after which verbal/oral informed consent was obtained from individual patient. The informed consent form and questionnaire were translated into Yoruba, the local language for majority of participants. Patients who do not understand English language were interacted with using the Yoruba version of the questionnaire. Back-translation was subsequently done to ensure response consistency. Patients were assured of confidentiality and anonymity of their responses, while they were informed that participation is entirely voluntary. The questionnaire was interviewed-administered to consented patients on every clinic day by the investigators. All the targeted eligible patients consented for participation and they were all enrolled and administered the questionnaire. Also, the medical records of DS-TB patients from January 2013 to December 2017 in each hospital were chronologically arranged according to the respective year, with relevant parameters retrieved and reviewed.

### Data analysis

Data obtained were sorted, coded and entered into the Statistical Package for Social Sciences (SPSS) version 23 for analysis. Descriptive statistics including frequency and percentage were used to summarise the data for prospective and retrospective cohorts. Treatment outcome rates for the retrospective cohort, including cure rate, treatment failure and default rate, as well as mortality or death rate were determined as total number each of TB patients who were cured, failed treatment, defaulted and died divided by the total number of patients who were commenced on TB treatment, multiply by 100 (e.g. cure rate = number of DS-TB patients cured ÷ total number of DS-TB patients placed on TB treatment × 100). Subsequently, treatment success rate was calculated as the sum total of all the patients who were cured and completed treatment (i.e. successful treatment) divided by the total number of patients who were commenced on TB treatment (i.e. successful and unsuccessful treatment) multiply by 100. Transferred out TB patients were excluded as component of unsuccessful treatment since they were not placed on any form of TB treatment. Person Chi-square (χ2) was used to investigate association between relevant patients’ characteristics and those with or without successful TB treatment outcome. Priori level of significance was set at *p* < 0.05.

## Results

### Prospective participants

All the participants enrolled from both hospitals within the study period consented to partake in the study, giving a response rate of 100%.

#### Socio-demographic characteristics

Out of the 140 DS-TB patients who were administered the questionnaire, 40 (28.6%) were from UCH and 100 (71.4%) from GCHJ. Seventy seven (55.0%) were males and 63 (45.0%) were females. Most (63; 45.0%) patients were within the ages of 18–34 years, while secondary education was highest (66; 47.1%). Fifty-one (36.4%) of the DS-TB patients were in the intensive phase of treatment, while 89 (63.6%) were in the continuous phase (Table l). Of the presenting symptoms reported by patients at the onset of TB infection, cough was the highest manifestation in different combinations (140; 33.7%) Table 1.

**Table 1:** Socio-demographic and clinical characteristics (prospective participants)

Variable	Frequency	Percent
**Age (year)**		
18–34	63	45.0
35–54	56	40.0
≥ 55	21	15.0
**Sex**		
Male	77	55.0
Female	63	45.0
**Educational level**		
No formal education	6	4.3
Primary	17	12.1
Secondary	66	47.1
Tertiary	51	36.4
**Occupation**		
Trading	44	31.4
Artisan	32	22.9
Retiree	23	16.4
Student/unemployed	22	15.7
Public/civil servant	19	13.6
**Marital status**		
Single	49	35.0
Married	81	57.9
Separated	6	4.3
Widowed	4	2.9
**DOTs clinic attended**		
UCH	40	28.6
GCHJ	100	71.4
**Duration on TB treatment (month)**		
One	31	22.1
Two	20	14.3
Three	23	16.4
Four	19	13.6
Five	29	20.7
Six	18	12.9
**Family member on TB treatment**		
Yes	7	5.0
No	133	95.0
***If yes, the relationship***		
Siblings	3	2.1
Parents	2	1.4
Children	1	0.7
Niece	1	0.7
**Symptoms experienced at the onset of TB infection (*n*** = 416)*		
Cough	140	33.7
Weight loss	77	18.5
Fatigue	69	16.6
Low grade fever	44	10.6
Night sweats	38	9.1
Chest pain	25	6.0
Loss of appetite	23	5.5

*multiple response, DOTs = Directly Observed Therapy Short-course, *UCH* University College Hospital, *GJCH* Government Jericho Chest Hospital, *TB* Tuberculosis, *n* number

#### Knowledge of tuberculosis, modes of transmission and suggested preventive measure

A total of 137 (97.9%) patients knew that TB is a curable disease, with most (76; 55.5%) patients obtained the information from healthcare professionals, mostly nurses (42; 32.7%) and physicians (34; 24.8%), while pharmacists were not cited. Also, 128 (91.4%) knew that TB can be transmitted to another person, while 107 (46.1%) accurately cited coughing without covering the mouth as a principal mode of TB transmission. Covering of mouth when coughing (123; 49.6%) topped the list of suggested measures to prevent TB transmission, while 10 (4.0%) mentioned adherence to TB medications (Table 2).

**Table 2:** Knowledge of tuberculosis, mode of transmission and suggested preventive measures (prospective participants)

Variables	Frequency	Percent
**Can tuberculosis be cured? (*n*** **= 140)**		
Yes	137	97.9
No	1	0.7
Do not know	2	1.4
**If yes, the source of information (*n*** **= 137)**		
Nurses	42	30.7
Physician	34	24.8
Family and friends	22	16.1
Self-belief	21	15.3
Internet	11	8.0
Media (radio)	4	2.9
Education background	3	2.2
**Can tuberculosis be transmitted? (*n*** **= 140)**		
Yes	128	91.4
No	5	3.6
Do not know	7	5.0
**If yes, summary of common modes of TB transmission (*n*** = 232)*		
Coughing without covering the mouth	107	46.1
Sharing of cutleries, plates, cups etc.	46	19.8
Indiscriminate/careless spitting	23	9.9
Overcrowding	21	9.1
Through the air	20	8.6
Kissing	13	5.6
Singing, sneezing	1	0.4
Others e.g. sexual intercourse, use of sharp objects	1	0.4
**Suggested measures for prevention of TB transmission (*n*** = 248)*		
Covering mouth when coughing	123	49.6
Do not share any cutlery	65	26.2
Always avoid indiscriminate spitting	30	12.1
Ensuring proper ventilation	13	5.2
Taking drugs as prescribed	10	4.0
Observing personal hygiene	4	1.6
Do not share clothing	2	0.8
Avoiding sexual intercourse for some period during TB treatment	1	0.4
**Knowledge of medications to treat TB infection (*n*** **= 140)**		
Yes, Isoniazid, Rifampicin, Pyrazinamide and Ethambutol (HRZE)	6	4.3
Don’t know	134	95.7

*multiple response, *TB* Tuberculosis, *n* number

#### Anti-tuberculosis and adjunct medications taken by patients

All the DS-TB patients (51; 100%) in the intensive phase were prescribed quadruple combination of isoniazid (H), rifampicin (R), pyrazinamide (Z) and ethambutol (E), while the 89 (100.0%) patients in continuation phase were on the dual combination of rifampicin and isoniazid. The class of adjunct medications taken by the patients were haematinics (50; 52.1%), antibacterial (13; 13.5%), antiretroviral therapy (12; 12.5%), antihypertensive (6; 6.3%), antidiabetic (4; 4.2%), cough syrup (4; 4.2%), anticonvulsant (3; 3.1%), antipsychotic (2; 2.1%), antiasthmatic (1; 1.0%), and one (1.0%) mentioned herbal preparation.

#### Reasons for TB treatment non-adherence and side effects experienced with tuberculosis medications

Forty-nine (35.0%) indicated reasons for TB treatment non-adherence, while 91 (65.0%) gave no specific reason. Inaccessibility to healthcare facility (33; 55.0%) topped the list of reasons for TB treatment non-adherence. Other reasons mentioned included too much medications to take at once (10; 16.7%) and size of the tablet being taken (8; 13.9%) Table 3. Sixty-seven (47.9%) reported to have experienced side effect(s) with TB medications, while 73 (52.1%) did not. Of this, 37 (55.2%) reported the experienced reaction(s) to their physician, while 30 (44.8%) did not report (Table 3).

**Table 3:** Reasons for treatment non-adherence and experienced side effects (prospective participants)

Variables	Response, ***n*** (%)	
**Any reason(s) for TB treatment non-adherence**	Yes, 49 (35.0)	No, 91 (65.0)
If yes, specific reason(s) (n = 60)*		
Problem of accessibility to healthcare facility	33 (55.0)	
Too much medicines to take at once	10 (16.7)	
Size of the tablet being taken	8 (13.3)	
Dosing schedule/time of medication	6 (10.0)	
Fear of medication side effects	3 (5.0)	
**Did you experienced side effect(s) with the TB medication(s) since commencement?**	Yes, 67 (47.9)	No, 73 (52.1)
If yes, specific side effect(s) experienced (n = 78)*		
Coloured urine	31 (39.7)	
Allergic skin reactions	16 (20.5)	
Tiredness	16 (20.5)	
Neuropathy signs	8 (10.2)	
Increased appetite	4 (5.1)	
Increased heartbeat	3 (3.8)	
**Did you report the experienced side effect(s) to your physician? (*n*** **= 67)**	Yes, 37 (55.2)	No, 30 (44.8)
***If yes, what measure(s) was taken by the physician? (n = 37)***		
It was expected reaction(s)/side effect(s)	16 (43.2)	
Prescribed anti-allergy medicine(s)	5 (13.5)	
Prescribed haematinics	3 (8.1)	
Prescribed analgesics	3 (8.1)	
Prescribed antibiotics	2 (5.4)	
Changed my medication(s)	1 (2.7)	
Cannot remember the medication(s) prescribed	7 (18.9)	
***If No, why don’t you report the experienced side effect(s)? (n = 30)***		
Already informed of the side effect(s)	15 (50.0)	
I intended to report	6 (20.0)	
I knew it was the medication(s) that caused the side effect	5 (16.7)	
I purchased antiallergy chlorpheramine on my own	3 (10.0)	
I purchased a combined antifungal and allergic cream on my own	1 (3.3)	

*multiple response, *HRZE* Isoniazid (H), Rifampicin (R), Pyrazinamide (Z), Ethambutol (E), *TB* Tuberculosis, *n* number

### Retrospective participants

#### Treatment outcomes among the DS-TB patients managed between 2013 and 2017

Of the 2400 medical records of DS-TB patients reviewed between 2013 and 2017, a total of 2389 (99.5%) had the required information documented, comprising 934 (39.1%) from UCH and 1455 (60.9%) from GCHJ. Also, the case notes reviewed for each year were 431 (18.0%) in 2013; 523 (21.9%) in 2014; 393 (16.5%) in 2015; 516 (21.6%) in 2016 and 526 (22.0%) in 2017. Out of the 2389 eligible case notes, 2262 (94.7%) patients had sputum smear AFB results documented in their case notes before the commencement of TB treatment. This comprised 1596 (70.6%) who had positive AFB sputum smear, and 666 (29.4%) with negative AFB sputum smear but chest X-ray and clinical presentation suggestive of active TB infection. The reminder 127 (5.3%) had neither AFB sputum smear nor chest X-ray results recorded, and were considered as transferred out patients. There was no documentation on TB culture test in the case notes of the DS-TB patients reviewed. Also, of the 2389 patients, 2117 (88.6%) had their HIV status documented, with 427 (20.2%) who had HIV positive status, while 1690 (79.8%) were HIV negative.

Table 4 shows details of treatment outcomes between 2013 and 2017. Of the 2262 (94.7%) whose treatment outcomes were retrieved from the case notes, 1211 (53.5%) DS-TB patients were cured, 580 (25.6%) had their treatment completed, 240 (10.6%) defaulted, 54 (2.3%) failed treatment, while 177 (7.8%) deaths were recorded. The treatment success rate between 2013 and 2017 ranged from 77.4 to 81.9%, and overall 1791 (79.2%) had successful treatment outcome (cured + completed treatment) within the 5-year period. Associations between relevant socio-demographic characteristics and TB treatment outcomes are shown in Table 5. There were significant associations between sex (χ2 = 8.780, *p* = 0.003), HIV status (χ2 = 29.110, *p* < 0.001), DOT clinic attended (χ2 = 18.215, *p* < 0.001) and patients’ with or without successful treatment outcome. Treatment were significantly successful among males TB patients (57.4%) compared to their female counterparts (42.6%), *p* = 0.003, also TB patients with HIV negative status (82.2%) versus HIV positive status (17.8%), *p* < 0.001.

**Table 4:** Relevant patients’ characteristics and treatment outcome (retrospective participants)

	Treatment outcome, ***n*** (%)					
Variable	Cured	Treatment Completed	Defaulted	Failed treatment	Died	Transferred out
**Treatment outcome rate (%),** ***n*** **= 2262**	53.5	25.6	10.6	2.4	7.8	
**Age (year)**						
18–34	619 (51.1)	210 (36.2)	128 (53.3)	21 (38.9)	51 (28.8)	66 (51.9)
35–54	469 (38.7)	272 (46.9)	83 (35.6)	26 (48.1)	85 (48.0)	43 (33.9)
≥ 55	123 (10.2)	98 (16.9)	29 (12.1)	7 (13.0)	41 (23.2)	18 (14.2)
Total	1211 (100.0)	580 (100.0)	240 (100.0)	54 (100.0)	177 (100.0)	127 (100.0)
**Sex**						
Male	724 (59.9)	304 (52.4)	160 (66.7)	35 (66.8)	111 (62.7)	72 (56.7)
Female	487 (40.2)	276 (47.6)	80 (33.3)	19 (35.2)	66 (37.3)	55 (43.3)
Total	1211 (100,0)	580 (100.0)	240 (100.0)	54 (100.0)	177 (100.0)	127 (100.0)
**HIV status (*n*** **= 2117)**						
Positive	129 (11.5)	171 (30.4)	67 (30.5)	9 (18.0)	51 (31.9)	28 (27.2)
Negative	995 (88.5)	392 (69.6)	153 (69.5)	41 (82.0)	109 (68.1)	75 (72.8)
Total	1124 (100.0)	563 (100.0)	220 (100.0)	50 (100.0)	160 (100.0)	103 (100.0)
**DOT clinic attended**						
UCH	251 (20.7)	389 (67.1)	115 (47.9)	18 (33.3)	86 (48.6)	75 (59.1)
GCHJ	960 (79.3)	191 (32.9)	125 (52.1)	36 (65.7)	91 (51.4)	52 (40.9)
Total	1211 (100.0)	580 (100.0)	240 (100.0)	54 (100.0)	177 (100.0)	127 (100.0)
**Year of enrolment**						
2013	207 (17.1)	118 (20.3)	33 (13.3)	7 (13.0)	32 (18.1)	34 (26.8)
2014	286 (23.6)	104 (17.9)	56 (23.3)	9 (16.7)	38 (21.5)	30 (23.6)
2015	197 (16.3)	93 (16.0)	39 (16.2)	8 (14.8)	28 (15.8)	28 (22.0)
2016	285 (23.5)	98 (16.9)	52 (21.7)	10 (18.5)	41 (23.2)	30 (23.6)
2017	236 (19.5)	167 (28.8)	60 (25.0)	20 (37.0)	38 (21.5)	5 (3.9)
Total	1211 (100.0)	580 (100.0)	240 (100.0)	54 (100.0)	177 (100.0)	127 (100.0)

Treatment outcome rate was calculated as the total number each of TB patients who were cured, completed treatment, defaulted, failed treatment and died, divided by the total number of patients placed on TB treatment (Y), multiply by 100, where Y = 2262. Transferred out patients were not part of the calculation for treatment outcome since they were not on TB treatment. *HIV* Human Immunodeficiency Virus, *UCH* University College Hospital, *GJCH* Government Jericho Chest Hospital, *DOT* Directly Observed Therapy, on TB treatment. Level of statistical significance, *p* < 0.05, *n* number

**Table 5:** Association between relevant characteristics and treatment outcome (retrospective participants)

Variable	Successful TB treatment (cured + completed treatment), ***n*** (%)	Unsuccessful TB treatment (defaulted + failed treatment + died), ***n*** (%)	Treatment success rate (%)
**Age (year)**			
18–34	829 (46.3)	200 (42.5)	80.6
35–54	741 (41.4)	194 (41.2)	79.5
≥ 55	221 (21.3)	77 (16.3)	74.2
	χ2 = 5.612; *p* = 0.060		
**Sex**			
Male	1028 (57.4)	306 (65.0)	77.1
Female	763 (42.6)	165 (35.0)	82.2
	χ2 = 8.780; *p* = 0.003*		
**HIV status (n = 2117)**			
Positive	300 (17.8)	127 (29.5)	70.3
Negative	1387 (82.2)	303 (70.5)	82.1
	χ2 = 29.11; *p* = 0.000*		
**DOTs clinic attended**			
UCH	640 (35.7)	219 (46.5)	74.5
GCHJ	1151 (64.3)	252 (53.5)	82.0
	χ2 = 18.215; *p* = 0.000*		
**Year of enrolment**			
2013	325 (18.1)	72 (15.3)	81.9
2014	390 (21.8)	103 (21.9)	79.1
2015	290 (16.2)	75 (15.9)	79.5
2016	383 (21.4)	103 (21.9)	78.8
2017	403 (22.5)	118 (25.1)	77.4
	χ2 = 2.973; *p* = 0.562		
**Overall TB treatment success rate within the 5-year period (1791 ÷ 2262)**			**79.2**

Treatment success rate was calculated as number of successful TB treatment divided by the sum of successful TB treatment plus unsuccessful treatment, multiply by 100. *HIV*Human Immunodeficiency Virus, *UCH* University College Hospital, *GCHJ* Government Chest Hospital Jericho, *DOTs* Directly Observed Treatment Short-course, *Significance difference with Chi-square test, Level of statistical significance, *p* < 0.05, *n* number

## Discussion

In this study, we evaluated knowledge about TB and possible reasons for TB treatment non-adherence among prospective ambulatory DS-TB patients, as well as reviewed the trend in treatment outcomes for DS-TB outpatients managed within a 5-year period in two WHO certified TB-DOT centres. Our study revealed that nearly 98 and 91%, respectively, succinctly understood TB to be curable, as well as being a disease transmittable from one person to another. Previous studies had reported between 76 and 95% of TB patients who knew about the curable nature of TB [36–41]. Higher values of 96.3 and 97.6% about patients’ awareness of the curability of TB had also been reported in other studies [42, 43]. In addition, coughing without covering the mouth, sharing of cutleries and indiscriminate spitting by TB-infected individuals were copiously cited by patients in our study as common modes of TB transmission. This is consistent with previous studies where varying proportions, 25, 53.6 and 63.4% of their patients were reported to be aware of cough hygiene, as an important TB preventive measure [42–44].

Noteworthy to mention that, only 1.2% of the TB patients in our study expressed some level of misconceptions about TB preventive measures, such as short-term abstinence from sexual intercourse during TB treatment, as well as avoiding clothes sharing. This proportion is far less than 10.8, 15.5, and 22.7% in previous studies [39, 44, 45], where avoiding food and utensils were cited as modes of TB prevention. Surprisingly, 4.3% of the patients could correctly mentioned the current medications for treating TB infection, while only 4% cited adherence to TB medications as a measure to prevent TB transmission. The low proportion of patients with correct response in these regards is a concern that may underscore the need for concerned stakeholders, especially the National Tuberculosis and Leprosy Control Programme (NTBLCP) and TB primary care providers to step-up counselling and enlightenment efforts for TB patients, especially in the areas of core TB preventive measures and modes of transmission, of which adherence to TB medications is most essential. Though, our study did not directly assign score in quantifying patients’ knowledge about TB, rather we focused largely to explore the depth of patients’ understanding of some basic aspects in TB management. However, the perceived good knowledge of patients in some TB preventive measures and modes of transmission seems encouraging and may further emphasise the necessity for continuous public education and enlightenment on TB prevention, treatment and care among the patients. Incidentally, in our study, nurses and physicians were the healthcare providers who were largely cited as source of knowledge information about TB, with no mention of pharmacists. Amazingly, pharmacists are expected to be the core healthcare provider to dispense and counsel patients on their TB medication usage. This perhaps reveals that pharmacists might not have been in direct contact with TB patients at the DOT service point in the hospitals. Thus, a call for concern among relevant stakeholders in the pharmacy profession in Nigeria, of the need to ensure and encourage pharmacists to be more proactively engaged in TB care, whether in the hospital or community pharmacy setting.

Topmost of the reasons cited by patients for TB treatment non-adherence were inaccessibility to healthcare facility, perhaps in terms of travel costs for daily DOT at the clinic, and the idea of taking many anti-TB medicines at once. Lack of access to formal health services, and the consequent non-clinic attendance, as well as poor socio-economic status among many patients with TB have been reported in previous studies [46, 47], as key factors hindering continuous progress of DOT concept in enhancing TB treatment adherence. Generally, the two studied TB-DOT facilities largely rely on healthcare-facility or clinic-based DOT, in which TB patients report to the clinic on a daily basis (opening hours 8a.m to 2p.m, in most cases) for their daily dose of TB medications under the direct observation of the attending primary care provider, who monitor and record the TB dose taken. As a result, many TB patients asides from tackling other competing routine demands such as job schedule overlapping with clinic appointment time [46], may also face the burden of daily transportation/travel costs to the clinic. Nevertheless, the challenge of healthcare inaccessibility may be partly overcome through consideration of non-healthcare facility or clinic-based DOT, perhaps the community-based DOT, where the healthcare provider visit the TB patients in their community to deliver the DOT service [10]. Although, community-based DOT may involve extra costs to the institution and other supporting partners, however, evidence has shown that community or home-based DOT had higher rates of treatment success in terms of cure, treatment completion and 2-month sputum conversion, as well as having lower rates of mortality and unfavourable outcomes compared with health facility-based DOT [10, 26]. In addition, decentralization of TB-DOT service to peripheral facilities closer to the people may also be a vital option to improve access to TB treatment. Many developed and developing countries have embraced decentralization of TB-DOT services, with a positive report of increased access to TB care [10, 11, 48, 49]. The WHO and NTBLCP have also advocated that further strengthening and decentralization of TB services may be a way forward to achieve the WHO End TB strategy [48, 49]. In Nigeria, the major drawback to the TB-DOT decentralization advocacy, may be the lack of competent healthcare personnel at the peripheral facilities to deliver the DOT services [19, 33, 48]. Thus, government and other concerned stakeholders may need to step-up their political and financial commitments toward TB treatment and care, in order to achieve the third united nation sustainable development goals and the WHO End TB strategy of eradicating TB globally by the year 2030 target [1, 50]. More importantly, institution of appropriate support systems, specifically, material support including financial incentives such as transport subsidies or financial bonus to TB patients may be essential, to at least take care of the indirect costs that are incurred by patients when attending the daily DOT clinic. In addition, consideration of fixed dose combination (FDC) tablets for TB medications may be useful to overcome the issue of pill burden raised by the patients. The FDC for TB medications is now a conditional recommendation in the 2017 update of WHO TB treatment guidelines, for DS-TB patients [10, 11].

A sizeable proportion of the patients claimed to have experienced side effect(s) with their TB medications, but only 5% cited fear of medication side effects as a reason for TB treatment non-adherence. More than one-third of the patients reported that the side effects experienced were expected reactions which they have been pre-informed by their primary care physician. Thus, they probably do not consider such side effect(s) as a barrier to TB treatment adherence. The overwhelming positive response of patients on pre-knowledge information about expected medication side effects is noteworthy and commendable. Therefore, such counselling role and value-added services should be continuous and consistently done at every TB patient-provider encounters. However, our study finding in this regard is in contrast with report from previous studies stating that patients were not informed about side effects and what to do to counter it [51, 52]. Providing counsel on possible adverse drug events in language the patient best understand may be helpful in preparing patients towards better appreciation and commitment to their treatment. Typically, the healthcare providers and supporting staff working in the TB-DOT centres used to undergo periodic TB care-related training, as well as seminars organised either by the respective hospital or the funding partners, largely to enhance job performance and competence. This might have helped the TB primary care providers in the efficient discharge of their clinical roles and duties in TB care and management. In general, TB primary care providers should continuously explore the possible reasons for poor TB treatment adherence at every patient-provider encounters, thereby making effort to offer necessary assistance, especially psychological support through robust counselling session or peer group support, with a view to collectively enhance treatment adherence and outcome [52–54].

Precisely, 54% of TB patients evaluated between 2013 and 2017 were cured, and nearly one-quarter had treatment completed, with close to one-tenth deaths recorded. An overall treatment success rate of approximately 79% was achieved within the 5-year period reviewed, which is about 12% below the WHO-defined target of 90% for new TB cases [1, 7, 10]. The treatment success rate noted in our study is higher than the values recorded in some developing countries [55–58], while studies conducted in high-income countries reported a higher treatment success rates [7, 26]^.^ Varying TB treatment success rates ranging from 34 to 85% in the low- and middle-income countries have earlier been reported [1, 50]. In addition, the default rate of about 10% obtained in our study is three times the WHO target of 3% default rate among TB patients [7, 10], but the value is still lower than that reported in previous studies in Nigeria [57, 58]. Also, the TB default rates in other studies conducted in South Africa [59] and Brazil [60] reported higher rates of default than found in our study. Thus, as previously suggested, there may be a need for institution of appropriate support systems, which may include material, structural and psychological supports [61] for all categories of DS-TB patients, as this may go a long way to relief the patients’ disease burdens, with greater likelihood of facilitating optimal commitment to TB treatment, and subsequently, there may be improved treatment outcomes and success rate.

In our study, we observed that 8.6% among the prospective DS-TB participants reported antiretroviral therapy (ART) as adjunct medications taking alongside the core anti-TB medications. Although, we may not be able to directly assume this percent prevalence as proportion who may genuinely have HIV positive status. The use of ART drugs with anti-TB medications may perhaps indicate a greater possibility that the concerned patients may be managing or treating a TB co-infected HIV infection. In addition, a value of 20.2% HIV positive status documented in the medical records of DS-TB patients in the retrospective cohort may seem more reliable than the indirect prediction of patient’s HIV status from the self-report mentioning of antiretroviral medications taking by the patients. Nevertheless, a carefully considered future study to further explore the precise prevalence of TB/HIV co-infection may be necessary, in order to make a far-reaching conclusion. The HIV prevalence observed among the DS-TB patients in the retrospective cohort is lower that the HIV prevalence of between 27.2 and 61% reported among TB patients in Eastern and Southern Africa countries [53, 59, 62]. It is noted that TB treatment outcomes was significantly successful among TB patients with HIV negative status, compared to the HIV positive counterparts. Poor treatment outcome among HIV co-infected TB patients has been corroborated by other studies, where HIV co-infection was found to increase the chance of unsuccessful treatment outcome among TB patients [57, 63–65]. In general, the low treatment success rates perhaps further reiterates the necessity for concerned stakeholders in tuberculosis control in LMICs including Nigeria, to step-up efforts at ensuring institutionalization of functional and robust TB patients’ support systems, increased advocacy and enlightenment on TB control, as well as consistent availability of anti-TB and relevant adjunct medications at the TB-DOT service centres.

Despite the useful information from our study, the following limitations are worthy of mentioning. This includes the possibility of documentation bias that may arise from patients’ medical records. In the studied facilities, all the DS-TB patients were placed on the same standard 6-month short-course regimen of 2HRZE/4HR, with no discrimination into category 1 (new TB cases) or category II (retreatment TB cases). This was consistent all through the period of review, and this treatment approach conforms to the 2017 update of the WHO TB treatment guidelines [10]. Also, the cross-sectional nature of our study may not concisely permit the establishment of a causal relationship, while the inherent limitation(s) such as recall bias from the self-report measure [37] may not be totally excluded. Nevertheless, the use of non-judgemental and non-threatening question-items may probably allow for a sincere opinion among the patients. In addition, the representativeness of sampled population, as well as conduct of the study in two WHO-certified TB-DOT centres may perhaps ensure collection of a more reliable data on TB management, thus a useful strength for our study. Another limitation may be linked to the non-availability of the reasons for treatment non-adherence among the DS-TB patients whose case notes were retrospectively reviewed. Also, patients in the prospective cohort were not explored on other useful patients’ characteristics such as living condition, monthly income, residence area/distance from the DOT facility, lifestyles especially smoking and alcohol intake, which were largely considered to be outside the scope of our study objectives. We focused generally on gaps that may not have been concisely captured in the previous related studies. In addition, the prospective patients were not follow-up to explore their treatment outcomes. These limitations may therefore need to be carefully considered when making generalisation about our study findings.

## Conclusions

It can be concluded that knowledge about TB among the prospective DS-TB patients is relatively high, especially with respect to common modes of TB transmission and preventive measures, but a sizeable number lacks the understanding of ensuring optimal TB medication-adherence to prevent TB transmission. Inaccessibility to healthcare facility largely accounts for TB treatment non-adherence. Treatment outcomes within the 5-year period show that nearly half were cured, while almost one-tenth died. Overall treatment success rate of 79% achieved is about 12% below the WHO-defined target. There is generally a need for concerned stakeholders to step-up efforts in ensuring consistent TB enlightenment, while improving access to TB care is essential, perhaps by instituting necessary support systems including financial incentives/subsidies for TB patients generally. Also, the TB primary care provider should consistently re-evaluate the possible reason(s) for TB treatment non-adherence during provider-patient encounters and endeavour to offer essential psychological support through value-added counselling, with a view to increase treatment outcomes and success rate.

## Supplementary Information

**Additional file 1.** Questionnaire for the prospective cohort

## Data Availability

The datasets used and/or analysed during the current study are available from the corresponding author on reasonable request.

## Abbreviations

TB: Tuberculosis
DS-TB: Drug sensitive tuberculosis
DOTS: Directly Observed Treatment Short-course
IRB: Institution Review Board
ERC: Ethics Review Committee
NHREC: National Health Research and Ethics Committee
HRZE: Isoniazid-H, Rifampicin-R, Pyrazinamide-Z and Ethambutol-E
AFB: Acid Fast Bacilli
MDR-TB: Multidrug Resistance Tuberculosis
LMICS: Low and middle income countries
WHO: World Health Organisation
NTBLCP: Tuberculosis and Leprosy Control Programme
HIV: Human Immunodeficiency Virus
ART: Antiretroviral Therapy
SPSS: Statistical Package for Social Sciences
UI: University of Ibadan
UCH: University College Hospital
GCHJ: Government Chest Hospital, Jericho
