# Supplementary material for: Knowledge about tuberculosis, treatment adherence and outcome among ambulatory patients with drug-sensitive tuberculosis in two directly-observed treatment centres in Southwest Nigeria
Source: BMC Public Health. 2021 Apr 7;21:677. doi: 10.1186/s12889-021-10698-9 (PMC8028094; doi:10.1186/s12889-021-10698-9)
Supplement: Supplementary file 1 — Additional file 1. Questionnaire for the prospective cohort [file 12889_2021_10698_MOESM1_ESM.doc]

**ADDITIONAL FILE 1- QUESTIONNAIRE FOR PROSPECTIVE COHORT**

**INTRODUCTION**

Dear Sir/Ma,

I am a Masters student in the Department of Clinical Pharmacy and Pharmacy Administration, University of Ibadan. I am carrying out a study on “KNOWLEDGE ABOUT TUBERCULOSIS, MEDICATION ADHERENCE AND TREATMENT OUTCOMES AMONG DRUG-SENSITIVE TUBERCULOSIS PATIENTS IN AMBULATORY CARE SETTINGS”. I would appreciate if you could spare some time to answer the question-items below. Confidentiality of response and your anonymity are highly assured.

**Tick as appropriate**

Do you consent/agree to partake in this study? Yes ( ) NO ( )

Thank you.

**SECTION A:** **SOCIO-DEMOGRAPHICS AND CLINICAL CHARACTERISTICS**

**Tick or fill in your response appropriately**

**SOCIO-DEMOGRAPHIC INFORMATION**

1. Age:
2. Sex: Male { } Female { }
3. Marital status: Single { } Divorced { } Widowed { } Married { } Separated ( }
4. Level of Education: No formal education { } Primary { } Secondary { } Tertiary { }
5. Occupation: Student/unemployed { } Trading { } Artisan { } Public/Civil Servant { } Retiree ( ) Others, specify

**PATIENT’S CLINICAL CHARACTERISTICS**

1. How long have you been on tuberculosis treatment?
2. Is any member of your family having tuberculosis? YES{ } NO{ } I don’t know{ }
3. If YES, please specify the relationship
4. What are the symptoms you experienced at the onset of tuberculosis? Please tick as appropriate.

Cough { } Low grade Fever { }

Night sweats { } Loss of appetite { }

Weight loss { } Fatigue { }

Others, please specify

**SECTION B: ASSESSMENT OF PATIENTS’ KNOWLEDGE ABOUT TUBERCULOSIS, MODE OF TUBERCULOSIS TRANSMISSION AND SUGGESTED PREVENTIVE MEASURES** (Kindly, indicate your opinion to the statements below as it applies to you).

1. In your own opinion, can tuberculosis be cured? YES { } NO { }
2. If YES, please specify the source of your information
3. In your own opinion, can tuberculosis be contacted from another person? YES{ } NO{ }
4. If YES to question 12, what are the common modes of tuberculosis transmission?
5. If YES to question 13, what measures can be taken to prevent tuberculosis transmission?
6. What medications are you currently taking to treat tuberculosis?
7. Is there any other medication(s) you are concurrently taking? YES { } NO { }
8. If YES to question 16, kindly mention these other medication(s) aside from your regular anti-tuberculosis medications.
9. How long have you been taking these other medications with your tuberculosis medications?

**SECTION C: EVALUATION OF BARRIERS TO TUBERCULOSIS MEDICATION ADHERENCE OR TREATMENT, SIDE EFFECTS EXPERIENCED WITH ANTI-TUBERCULOSIS MEDICATIONS AND REPORT OF SIDE EFFECTS EXPERIENCED**

1. Is there any reason(s) that is/are preventing you from taking anti-tuberculosis medications as prescribed by your physician YES ( ) NO ( )
2. If YES to question 19, kindly indicate your opinion to the statement listed below in relation to possible reasons that may be preventing you from taking your tuberculosis medication/treatment as prescribed. (**You may tick more than one options as it applies to you**)

Taking more than 2 to 3 tablets at once. (polypharmacy) ( )

Inconvenient dosing time of the medication ( )

Side effects experienced ( )

Size of tablets consumed ( )

Accessibility of health facility ( )

Fear of addiction ( )

Stigma ( )

No reason ( )

Others, kindly specify____________________________________________

1. Have you experienced any side effect(s) since you started taking your anti-tuberculosis medications? YES{ } NO{ }
2. If YES to question 21, please mention the side effect(s) you have experienced.
3. Did you report these side effects to your doctor/physician? YES { }NO { }
4. If YES to question 23, how did the doctor/physician intervene in resolving the problem?
5. Have you had cause to stop medications taken for other conditions because of your tuberculosis medications? YES { } NO { }
